# Editorial: The Biology of Language Under a Minimalist Lens: Promises, Achievements, and Limits

**DOI:** 10.3389/fpsyg.2021.654768

**Published:** 2021-02-22

**Authors:** Antonio Benítez-Burraco, Koji Fujita, Koji Hoshi, Ljiljana Progovac

**Affiliations:** ^1^Department of Spanish, Linguistics and Theory of Literature (Linguistics), Faculty of Philology, University of Seville, Seville, Spain; ^2^Department of Human Coexistence, Graduate School of Human and Environmental Studies, Kyoto University, Kyoto, Japan; ^3^Faculty of Economics, Keio University, Yokohama, Japan; ^4^Linguistics Program, Department of English, Wayne State University, Detroit, MI, United States

**Keywords:** language, biology, minimalism, brain, cognition, evolution

Language can be approached from a variety of perspectives, e.g., philosophical, social, historical, psychological, biological, or physical, and it has been investigated from those perspectives throughout the history of language study. Among them, the generative enterprise was launched by Noam Chomsky in the 1950s (Chomsky, 1975), identifying language as a biological object of study. While the biological nature of language was clearly illustrated in Lenneberg's (1967) seminal work, linguistic theorizing including generative grammar has been too “linguistics-specific” to marry biology. However, with the advent of the minimalist program (MP) for linguistic theory advocated by Chomsky (1993), the view toward the architecture of language has drastically changed in the field. In MP, the core of language as a mind/brain-internal system is regarded as a computational mechanism that generates hierarchically structured expressions to link them to the sensorimotor (SM) and conceptual-intentional (C-I) systems. The computational mechanism is a syntactic combinatorial operation called Merge, which recursively combines lexical items as conceptual atoms or already constructed syntactic objects to yield new syntactic objects. Thanks to such radical simplification of the architecture of language in theory, MP has paved the way toward exploring how to link language with biology in the context of biolinguistics (Jenkins, 2000), even though there are still significant challenges for the linking (Poeppel and Embick, 2005; Poeppel, 2012).

Almost 30 years have passed since the appearance of MP in Chomsky (1993) and a lot of research has been carried out both within the linguistics proper and its related fields thus far, and we think it is about time to evaluate whether and/or to what extent the study of the biology of language has been furthered and deepened in the context of the minimalist program, critically examining its promises, achievements and limits from a multitude of angles. In keeping to this goal of our Research Topic in the current volume, the contributors focus on aspects related to one or more of the following broad themes in the study of the biology of language under a minimalist lens: (i) how (knowledge of) human language is to be characterized; (ii) how (knowledge of) human language develops (ontogeny of language); (iii) how (knowledge of) human language is put to use; (iv) how human language is implemented in the brain; (v) how human language evolved (phylogeny of language) (the questions raised in e.g., Chomsky, 1986; Jenkins, 2000, among others).

With respect to the nature of knowledge of human language, Haspelmath takes issue with the traditional view in generative grammar that the building blocks of languages (features, categories, and architectures) are part of an innate blueprint for human language, arguing that they are to be derived from convergent cultural evolution, which is in fact more in line with the minimalist tenet of attributing as few domain-specific elements as possible to what he calls *human linguisticality*, or the biological capacity for language. He also emphasizes the importance of exploring human linguisticality from the perspective of Greenbergian approach to comparison given the structural uniqueness of languages with respect to lexicon, phonology, and morphosyntax. Furthermore, Progovac points out the need for sorting out what to keep and what to discard from among the fundamental assumptions in the current version of the minimalist program. Adopting a gradualist approach to the evolution of syntax, she argues that while binarity and syntactic hierarchy of projections are stable and useful postulates for biolinguistic considerations, the claim that Merge *per se* yields infinite recursion subsuming Move is harmful from the standpoint of both linguistic analysis and language evolution considerations. In addition, Gil and Shen address the relation between cognition and grammar by focusing on three different phenomenological domains (compositional semantics, metaphors and schematological hybrids), arguing that there are two kinds of cognitive structures, symmetric and asymmetric cognition, and that the latter is derived from the former with the introduction of asymmetric thematic-role assignment in grammar. They also claim that the distinction between symmetric non-grammatical and asymmetric grammatical cognition manifests itself in phylogeny, ontogeny, and the architecture of human cognition.

Concerning the ontogeny of human language, addressing first language acquisition in the context of minimalism, Goodluck and Kazanina make a case for the superiority of drawing on Merge and interface conditions/constraints such as working memory capacity in accounting for the contrast between children and adults with respect to word order and phrase structures, pronominal structures and long-distance dependencies. In their opinion, among the advantages of this view is that the theory of child language acquisition does not have to account for the unlearning of an incorrect grammar on the way to adult language.

With regard to how (knowledge of) language is put to use, addressing the issue of code-mixing in both neuro-typical and neuro-atypical speakers/signers, Aboh argues that code-mixing receives a natural explanation by interaction between an innate cognitive process of what he calls *recombination* as an instance of general Merge and executive functions in the brain which are responsible for vocabulary insertion. It is claimed that recombination allows language learners to select relevant linguistic features from heterogeneous inputs to yield new syntactic objects for code-mixing in hybrid grammars.

Concerning the issue of how (knowledge of) language is implemented in the brain, based on a fMRI study on sentence processing of nested and cross-serial dependencies, Tanaka et al. argue that their new concept of “Merge-generability,” i.e., whether the structural basis for a given dependency is provided by Merge, holds a key to a better understanding of the nature of human language characterized by strong generative capacity (Chomsky, 1965). They demonstrate the prominent localized activation in the left frontal cortex as well as the left middle temporal gyrus and angular gyrus in response to Merge-generable dependencies, providing evidence for the specialization of these brain regions for syntactic processing. Furthermore, based on event-related potential (ERP) studies, Gallagher tests, and offers evidence for, the minimalist program (MP) prediction that organisms that possess the faculty of language (FL) cognitively process “language-like systems” in a qualitatively distinct fashion, defining language-like systems in terms of recursion criteria. He points out that processing language-like systems with recursion such as certain domains of mathematics and music will crucially elicit the common language-related ERPs [the left-anterior negativity (LAN), N400, and P600] on a par with language.

Finally, regarding how (knowledge of) human language evolved in our species, there has been a debate over the saltationist vs. gradualist view of phylogeny of language. From a perspective of paleoanthropology, Tattersall espouses the non-gradualist/punctuationist view of language evolution in support of the position advocated in the minimalist program (e.g., Bolhuis et al., 2014). He argues that modern symbolic human behavior patterns and cognition emerged suddenly in a short period of time, whereas he casts doubt on the claim that externalization of I-language came after internalization of it in the hominin evolution of language. On the other hand, in favor of the gradualist view of language evolution, Corballis takes issue with the fundamental tenet of the minimalist program that unbounded generativity of I-language is due to Merge, which is unique to our species, *Homo sapiens*. Instead, he argues that such a property of I-language derives from our ability of mental travel in time and space, or more broadly our ability of imagination, which is also unbounded and recursive in generativity and is shared with non-human animals that move, although the degree of power has been expanded in our species. Miyagawa and Clarke put forth yet another version of gradualist or incremental approach to the emergence of an infinite, recursive combinatorial operation of Merge. They argue that apparent simple cases of compositionality as observed in non-human primate call combinations of the Old World monkeys are implemented by means of what they call “a dual-compartment frame” rather than Merge and suggest that the dual-compartment frame may have served as an input to Merge in the evolution of human language. Also in line with the gradualism, while addressing the issue of the evolvability of words in the framework of the minimalist program, Clark considers what needed to evolve for the emergence of words in human language, pointing out that, how lexical items and the lexicon evolved is especially poorly understood. In proposing what properties lexical items have and what determines these properties, he claims that the pointers and packaging approach to the structure of lexical entries (Glanzberg, 2018) can prove to be illuminating in exploring the evolution of words in our species. From a broader perspective, addressing current minimalist biolinguistic and usage-based approaches as the two main theoretical frameworks for the evolution of language, Pleyer and Hartmann point out that, unlike the traditional views in each approach, recent developments in each of the two approaches have seen more convergences than differences. They argue that the two approaches are getting closer in terms of four contentious issues: modularity/domain-specificity, innateness/development, biological/cultural evolution, and knowledge of language/its description. Lastly, in connection with the issue of phylogeny of human language as it is concerned with historical language change in cultural evolution, Ceolin et al. statistically demonstrate that less visible taxonomic traits such as syntactic parameters modeled within the generative biolinguistic framework provide insights into deep-time language history as a new tool of phylogenetic linguistics, contrary to long-standing assumptions in the field.

Needless to say, more interdisciplinary research is called for to pin down the biological nature of language, and it is hoped that our Research Topic in this volume will provide a stimulating trigger for more active collaborative research in the various fields relevant for the biology of language, going beyond the current minimalist program into the future.

## Author Contributions

AB-B, KF, KH and LP conceived, wrote, and approved the manuscript. All authors contributed to the article and approved the submitted version.

## Conflict of Interest

The authors declare that the research was conducted in the absence of any commercial or financial relationships that could be construed as a potential conflict of interest.

## References

[B1] Bolhuis J. J. Tattersall I. Chomsky N. Berwick R. C. (2014). How could language have evolved? PLOS Biol. 12:e1001934. 10.1371/journal.pbio.1001934 25157536 PMC4144795

[B2] Chomsky N. (1965). Aspects of the Theory of Syntax. Cambridge, MA: MIT Press. 10.21236/AD0616323

[B3] Chomsky N. (1975). The Logical Structure of Linguistic Theory. New York NY: Plenum.

[B4] Chomsky N. (1986). Knowledge of Language: Its Nature, Origin, and Use. New York, NY: Praeger.

[B5] Chomsky N. (1993). A Minimalist Program for Linguistic Theory. MIT Occasional Papers in Linguistics 1. Cambridge, MA: MIT Working Papers in Linguistics.

[B6] Glanzberg M. (2018). “Lexical meaning, concepts, and the metasemantics of predicates,” in The Science of Meaning: Essays on the Metatheory of Natural Language Semantics, eds Ball D. Rabern B. (Oxford: Oxford University Press), 197–225. 10.1093/oso/9780198739548.003.0007

[B7] Jenkins L. (2000). Biolinguistics: Exploring the Biology of Language. Cambridge: Cambridge University Press. 10.1017/CBO9780511605765

[B8] Lenneberg E. H. (1967). Biological Foundations of Language. New York, NY: Wiley. 10.1080/21548331.1967.11707799

[B9] Poeppel D. (2012). The maps problem and the mapping problem: two challenges for a cognitive neuroscience of speech and language. Cogn. Neuropsychol, 29, 34–55. 10.1080/02643294.2012.710600 23017085 PMC3498052

[B10] Poeppel D. Embick D. (2005). “Defining the relation between linguistics and neuroscience,” in Twenty-First Century Psycholinguistics: Four Cornerstones, ed Cutler A. (Mahwah, NJ: Lawrence Erlbaum), 103–118.

